# Failure Mechanism and Optimization of Metal-Supported Solid Oxide Fuel Cells

**DOI:** 10.3390/ma16113978

**Published:** 2023-05-26

**Authors:** Pengxuan Du, Jun Wu, Zongbao Li, Xin Wang, Lichao Jia

**Affiliations:** 1 School of Materials Science and Engineering, Wuhan Textile University, Wuhan 430200, China; 2 School of Materials Science and Engineering, Huazhong University of Science and Technology, Wuhan 430074, China

**Keywords:** MS-SOFCs, failure mechanism, optimization method

## Abstract

A solid oxide fuel cell (SOFC) is a clean, efficient energy conversion device with wide fuel applicability. Metal-supported solid oxide fuel cells (MS-SOFCs) exhibit better thermal shock resistance, better machinability, and faster startup than traditional SOFCs, making them more suitable for commercial applications, especially in mobile transportation. However, many challenges remain that hinder the development and application of MS-SOFCs. High temperature may accelerate these challenges. In this paper, the existing problems in MS-SOFCs, including high-temperature oxidation, cationic interdiffusion, thermal matching, and electrolyte defects, as well as lower temperature preparation technologies, including the infiltration method, spraying method, and sintering aids method, are summarized from different perspectives, and the improvement strategy of existing material structure optimization and technology integration is put forward.

## 1. Introduction

With the rapid economic development of countries around the world, the problem of energy shortages has become increasingly prominent. The energy conversion efficiency of thermal power generation from traditional fossil energy is low (30–38%), and a large amount of dust, SO_2_, and other pollutants are generated, seriously wasting resources and polluting the environment [1,2,3].

Solid oxide fuel cells (SOFCs) continuously and directly convert chemical energy in fuel into electric energy, representing a highly interesting technology in the field of clean energy. SOFCs have high modularity, have a wide fuel application range, are free from the limitation of the Carnot cycle, and have high energy conversion efficiency. The improvement in the energy conversion efficiency effectively reduces carbon emissions, which is a technical goal for competing countries around the world. After decades of efforts by researchers, SOFCs have reached high technical level in key materials, structural design, and preparation processes; have realized the development from electrolyte-supported SOFCs (ES-SOFCs) to anode-supported SOFCs (AS-SOFCs); and have successfully reduced the operating temperature of SOFCs. The recent operating temperature of 550–750 °C makes it possible to use metal as supports.

**Figure 1:**
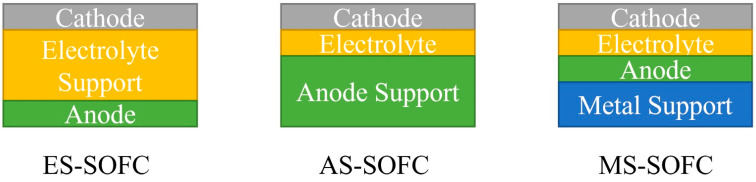
Schematic representation of ES-SOFC, AS-SOFC, and MS-SOFC.

Traditional ES-SOFCs and AS-SOFCs use ceramic or cermet materials as supports to provide mechanical strength, which is costly and prone to structural failure when subjected to rapid thermal cycling or mechanical shock, seriously hindering the commercialization of SOFCs [4]. Metal-supported solid oxide fuel cells (MS-SOFCs) use inexpensive, strong, and easily processed porous metals as supports, and the electrochemically active layer is directly applied to the support, providing significant benefits in terms of volume, cost, manufacturing, durability, and operation (Figure 1). Compared to traditional SOFCs, MS-SOFCs have higher performance at lower temperature [5]. This is due to the high ohmic impedance of conventional SOFCs, which usually require increased operating temperature to achieve appreciable performance [6,7].

Metal-supported solid oxide fuel cells (MS-SOFCs) can be prepared at lower costs and more compact volumes than traditional ceramics by using inexpensive, strong, and easily processed metals. Due to the good thermal conductivities and mechanical properties of the metal substrate that occupies most of the volume of MS-SOFC, the cell has good thermal shock resistance and excellent quick start ability, reducing the starting time of traditional SOFCs to ten or fewer minutes, accelerating the development of mobile vehicles and other fields [8,9]. This kind of good thermal shock resistance makes MS-SOFC also innovative in operation. In response to the fluctuating power requirements of many potential applications of SOFC power systems, Tucker et al. [10] proposed a method of dynamic temperature operation of SOFC systems, which enables SOFC performance to be more closely matched with application requirements at a lower cost. Moreover, compared to conventional SOFC, MS-SOFC is more suitable for providing power through a simple direct flame configuration when no other method is available [11]. In addition, the metal-supported solid oxide electrolytic cell (MS-SOEC), which is the inverse working process of MS-SOFC, also has similar advantages. It is desirable to use variable sources such as wind and solar to provide renewable electricity to drive the electrolysis, but this can result in transient operation of the electrolysis unit and produce rapid thermal transients that may be harmful to conventional SOEC [12]. In this case, the good thermal shock resistance of MS-SOEC is critical and may have an advantage over traditional SOEC [5]. However, it should be noted that the degradation rate of the metal-supported cells in SOEC mode is significantly faster than that in SOFC mode due to the coarsening of the fuel electrode catalyst, Cr poisoning of the air electrode catalyst, the oxidation of metal support, and so on [13]. In this regard, researchers have innovated the performance of MS-SOEC by pre-oxidation, optimizing catalysts, coatings, and so on [13,14]. Since this paper mainly studies SOFC mode, we will not elaborate too much here.

MS-SOFCs have many commercial advantages; however, their development faces many challenges. In addition to various problems, including carbon deposition, sulfur poisoning, and catalyst coarsening, the use of metals generates further problems, such as high-temperature oxidation, interdiffusion of support and anode elements, cathodic chromium poisoning, thermal matching, and electrolyte defects. These problems limit the long-term stability of MS-SOFCs and seriously hinder their commercialization.

By considering these problems, we summarize the existing problems and failure mechanisms of MS-SOFCs from different aspects and propose future improvement suggestions based on the existing optimization methods.

## 2. Failure Analysis

MS-SOFCs have excellent commercialization prospects; however, their technical development faces many challenges. In addition to the problems of carbon deposition, catalyst agglomeration coarsening, and other common problems, the use of metal supports leads to various issues, including high-temperature oxidation, cation interdiffusion problems, thermal matching, and electrolyte defects.

### 2.1. High-Temperature Oxidation Problem

High-temperature oxidation refers to the corrosion of metals caused by the reaction of metals with oxygen to form oxides at high temperature [15]. The high-temperature oxidation of MS-SOFCs mainly occurs in the metal support, anode, and interconnect. As this paper mainly discusses the characteristic failures of MS-SOFCs, the oxidation of metal support is mainly discussed below. To date, the commonly used support materials are nickel-based (Ni, Ni-Fe, Ni-Mo, Ni-Al, etc.) and ferritic stainless steel-based materials (AISI 441, Crofer22 APU, ITM, etc.) [15,16,17,18,19,20].

**Figure 2:**
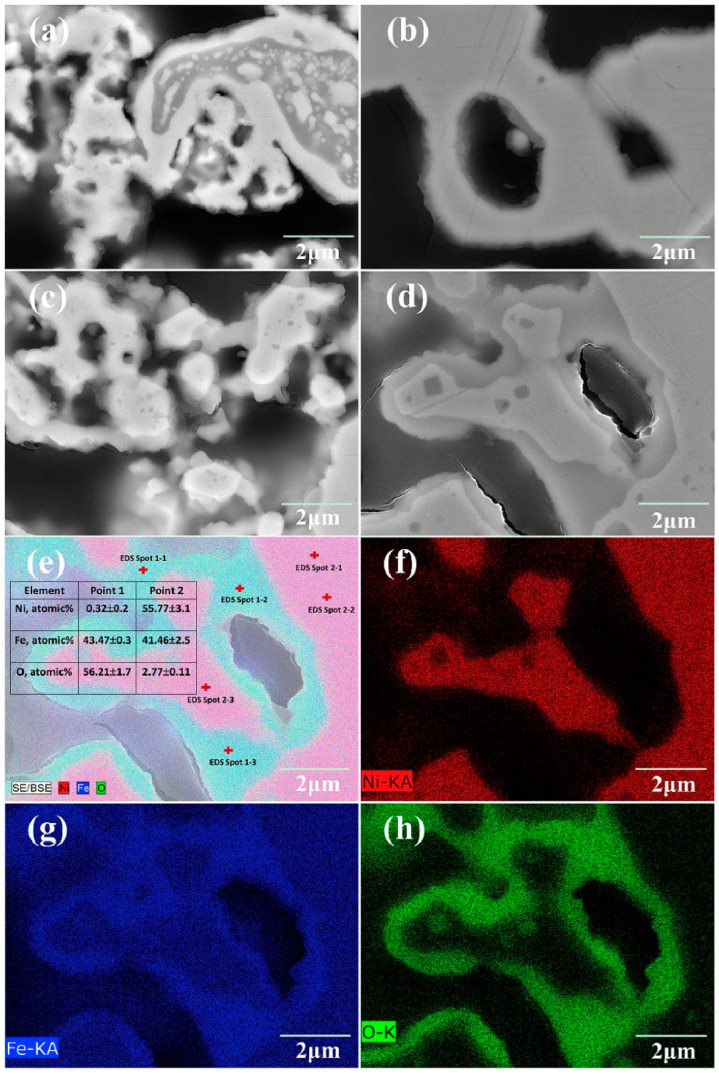
The scanning electron microscopy (SEM) images of the cross-sections of samples A (**a**) and B (**b**) after reduction and (**c**,**d**) after annealing in 75% H_2_/N_2_ (9/91)-25% H_2_O for 1000 h. For the area shown in (**d**), the energy-dispersive X-ray spectroscopy (EDS) point analysis and elementary map are shown in (**e**–**h**). Sample A is reduced at 750 °C for 10 h, and sample B is reduced at 1000 °C for 2 h [15].

Pure nickel has poorer oxidation resistance than ferritic stainless steel [21]. When the fuel gas in the anode chamber is interrupted during operation, nickel can be oxidized to NiO by the oxidation gas leaking into the anode chamber [22]. The addition of Fe to nickel-based support can improve the oxidation resistance of the support [22]. When oxidation occurs, Fe diffuses rapidly to the outer layer of the support and forms an oxide layer, preventing the further oxidation of internal Ni [22]. Zhu et al. [23] believed that the oxide layers formed on the binary Fe-Ni alloy are composed of an Fe_2_O_3_ top layer and an (Fe, Ni)_3_O_4_ spinel inner layer after 500 h of oxidation in 800 °C air condition. Xu et al. [15] have shown that an Fe_3_O_4_ oxide layer is formed in Ni-Fe support after long-term 75% H_2_/N_2_ (9/91)-25% H_2_O oxidation (Figure 2), and its conductivity (2.85 × 10^3^ S/cm) is sufficient for meeting the demand of MS-SOFCs.

**Figure 3:**
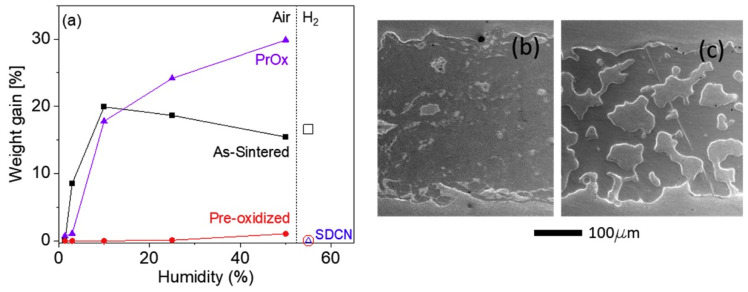
Breakaway oxidation of porous P434L stainless steel at 600 °C. (**a**) Humidity-dependence of the weight gain after 500 h in air (filled markers) or hydrogen/steam (open markers), for as-sintered (squares), pre-oxidized (circles), or coated (triangles) coupons. Pr_6_O_11−x_ (PrO_x_)-coated was exposed to air and Ni/Ce_0.8_Sm_0.2_O_2_ (SDCN)-coated was exposed to hydrogen/steam. Note that air with humidity in the range of ~1.5% to 50% was studied, but only 55% humidity was studied for hydrogen/steam. The markers for pre-oxidized and Ni-SDC samples in hydrogen/steam overlap. SEM images of polished cross-sections of porous P434L stainless steel after oxidation for 500 h in air with 25% humidity for (**b**) as-sintered and (**c**) pre-oxidized coupons without coating [28].

**Figure 4:**
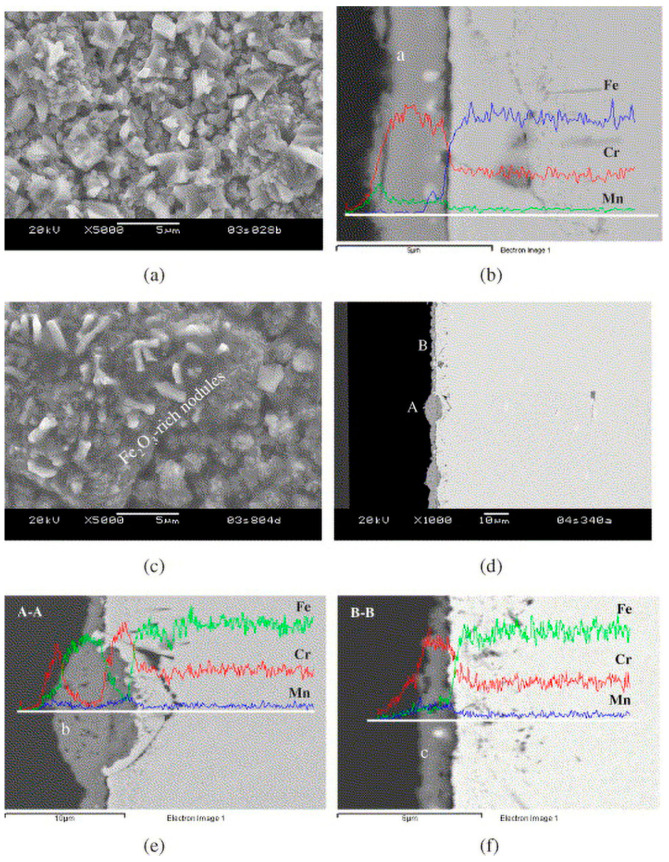
SEM observation of the scale on Crofer22 APU after isothermal oxidation at 800 °C for 300 h: (**a**) surface and (**b**) cross-section microstructures of the scale grown on the coupon exposed to moist air on both sides; (**c**) surface and (**d**) cross-section microstructures of the airside scale on the coupon simultaneously exposed to moist air on one side and moist hydrogen on the other; enlarged images from (**e**) area A and (**f**) area B in (**d**). The results of the EDS linear analysis on cross-sections are included [24].

When ferritic stainless steel is oxidized, the Cr in the substrate diffuses outwards and first forms a Cr_2_O_3_ layer on the surface. This compact chromium oxide film can effectively slow the oxidation rate, and it has excellent oxidation resistance. However, the oxidation behavior of ferritic stainless steel is still affected by many factors, including substrate composition, pore structure, and operating conditions; thus, special attention should be given to fabrication and operation [24,25,26,27,28,29,30,31]. When the chromium content of stainless steel is low (16–18%), it is not enough to form a protective chromium oxide layer [27]. The oxide layers of ferritic Fe-Cr alloys containing a small amount of Mn are composed of chromium oxide and manganese chromium spinel [28,29]. Mn is a spinel phase stabilizer that can increase the proportion of the spinel phase in the oxide layer [30]. Alloying elements, such as Ti, Si, and Nb, may reduce the kinetic rate of chromium oxide formation, and they are harmful to the formation of the oxide layer [30]. A porous structure with a high specific surface area accelerates the oxidation rate [27]. In addition, due to the slow diffusion rate of chromium at approximately 600 °C, a dense oxidation layer cannot be formed, and special attention should be given to the problem of breakaway oxidation [28]. This oxidation weight gain is an order of magnitude higher than that of normal oxidation (Figure 3). Moreover, research shows that there are certain differences in the morphologies and compositions of ferritic stainless steel oxidation in hydrogen and oxygen [24]. For ferritic stainless steel with a relatively low Cr content, the combined effect of hydrogen transport from the fuel side to the airside and the increase in water vapor on the airside seem to accelerate the oxidation and growth of Fe_2_O_3_ hematite-rich nodules on top of the oxide layer. Due to the large consumption of metal elements in the substrate of the nodule region, the oxide scale further penetrates into the metal substrate, leading to localized metal loss or attack; this abnormal oxide layer growth accelerates the erosion rate of the metal substrate (Figure 4) [24].

To date, to improve the oxidation resistance of stainless steel materials, it is often necessary to conduct pre-oxidation treatment to form a dense protective layer on the surface of the stainless steel substrate, thus inhibiting further oxidation of the internal substrate; the protective layer formed by the untreated substrate in the subsequent operation process is relatively loose, which may not play a good role in further inhibition, making it difficult to achieve a good oxidation resistance effect [28]. Introducing a protective coating is also a common, effective, and simple optimization method [29].

### 2.2. Cation Interdiffusion

Traditional catalyst materials for SOFCs easily diffuse and agglomerate during high-temperature sintering, thereby reducing the three-phase interface area and catalytic efficiency. Most MS-SOFC catalyst materials are the same as those of traditional SOFCs; thus, the above problems exist. In addition, the diffusion problem when using ferritic stainless steel as a support and interconnect material requires attention.

On the one hand, when the nickel-based anode contacts the support, Ni diffuses into the support during sintering or operation, austenitizing the ferritic support and causing thermal mismatch with other functional layers. Fe and Cr in the ferritic support diffuse into the Ni-based anode to form insulating oxides, reducing the catalytic and conductive properties [32,33].

**Figure 5:**
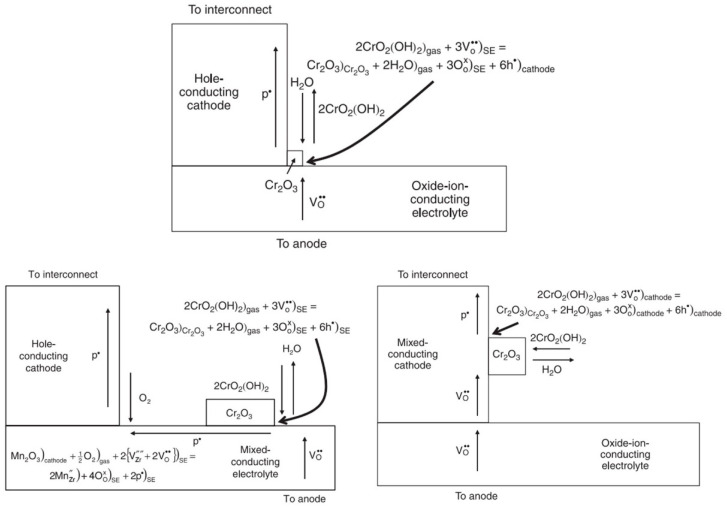
Electrochemical deposition of Cr_2_O_3_ on non-mixed conducting and mixed conducting electrolytes and electrodes [35].

On the other hand, the chromium oxide layer in the support and interconnect can poison the cathode through gasification, migration, and deposition (chromium-containing gas on the cathode electrochemical reduction to form Cr_2_O_3_), which is a common failure mechanism in MS-SOFCs. Cr poisoning is related to many factors. Temperature, humidity, and oxygen partial pressure affect the composition of chromium vapor, while the current, electrolyte, and electrode material characteristics affect the deposition of Cr [34,35,36]. When there is no current, random chemical deposition occurs, and the deposition rate is slow [35,36]. When there is a current, deposition preferentially occurs at the electrode–electrolyte three-phase interface, and the deposition amount increases with increasing current; the mixing of conductive electrolytes or electrodes can increase the electrochemical deposition area (Figure 5). In operating fuel cells, the oxide ion conductivity of lanthanum strontium cobaltite ferrite (LSCF) is much higher than that of lanthanum strontium manganite (LSM), and the deposition of Cr_2_O_3_ in non-manganese-containing electrodes is more evenly distributed and less localized, even observed near the interconnect [35,37]. Although many studies have been conducted, there are still great differences in the deposition mechanism of Cr. Some researchers believe that a combination of the electrochemical reduction mechanism and nucleation theory can better explain the Cr deposition phenomenon [36]. Huang et al. [38], for the first time, quantitatively determined the effect of Cr on the oxygen exchange kinetics of LSCF powder using in situ gas-phase isotope oxygen exchange. It is shown that the formation of secondary phases such as SrCrO_4_, Cr_2_O_3_, Cr-Co-Fe-O, and La-Co-Fe-O not only decreases the active sites for surface reactions but also alters the nearby stoichiometry of the LSCF matrix, thereby limiting surface oxygen transport [38].

The use of a barrier layer or coating can solve the diffusion and toxicity problems between stainless steel materials and anodes or cathodes [20,29]. Especially, anode and cathode catalyst coatings sintered at low temperature on the electrode skeleton can alleviate the coarsening caused by traditional high-temperature cosintering. Although nanoparticles with high specific surface areas are often coarsened during operation (especially nickel nanoparticles), the stability of an electrode with a coating can be improved by developing new materials, such as nickel–gadolinium-doped ceria (Ni-GDC), nickel–samaria-doped ceria (Ni-SDC), and nickel-free materials [39,40,41]. Precoarsening before operation can greatly improve stability; however, it comes at the expense of initial performance and should be used carefully [40].

### 2.3. Heat Matching Problems

In addition to the problems existing in the metal, the metal support brings hidden problems to the cell structure. On the one hand, the difference in the thermal expansion coefficient and sintering rate between metals and ceramics easily causes delamination and cracking during preparation and operation. Ni has good catalytic activity; however, face-centered cubic (FCC) pure Ni has a high thermal expansion coefficient (16∼17 × 10^−6^ K^−1^), which is difficult to match with other parts (10∼13 × 10^−6^ K^−1^). Therefore, FCC pure Ni is often combined with other elements (Fe, Al, etc.) to increase the thermal matching of materials [23,42,43,44,45]. The addition of Fe reduces the coefficient of thermal expansion of the support; with the increase in the mass ratio of Fe_2_O_3_, the coefficient of thermal expansion continues to decline. The coefficient of thermal expansion of NF50 is 13.7 × 10^−6^ K^−1^, achieving good matching with other functional layers [21]. Austenitic stainless steel with an FCC structure has stronger oxidation resistance, machinability, and mechanical strength than ferritic stainless steel; however, the thermal expansion coefficient (18∼20 × 10^−6^ K^−1^) is overly high, which is far less common than ferritic stainless steel. A wide variety of ferritic stainless steel with a body-centered cubic (BCC) structure has a thermal expansion coefficient of 11.5~14 × 10^−6^ K^−1^, which can be well matched with electrode and electrolyte materials and is the most widely used support material to date [46].

**Figure 6:**
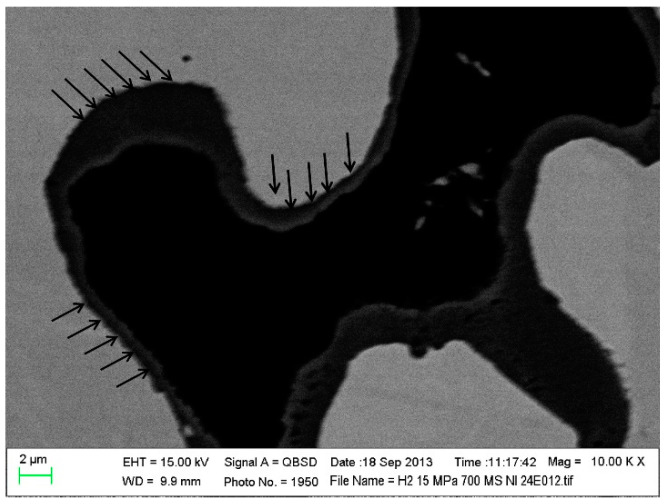
Micrograph from postmortem SEM analysis performed on the pre-oxidized MS-SOFC. The black arrows indicate the delamination between the oxide scale and the metal [48].

On the other hand, under the action of a constant temperature and load for a long time, the metal slowly undergoes deformation, which is a creep phenomenon [47,48,49]. The accumulation of creep does not lead to the failure of the metal material immediately, but it may produce creep deformation and cracks in the electrolyte, which accelerates the failure of the cell structure [49]. The element composition, operating temperature, time, atmosphere condition, stress, and oxidation behaviors of metals all influence the creep phenomenon. A small amount of Laves phase-forming elements Nb, W, and Si can significantly increase the creep resistance of ferrite steel at 700–800 °C, which is related to the solid solution strengthening and precipitation strengthening of Laves-phase particles [47]. The creep behavior caused by low temperature and stress can be ignored, and the creep rate often increases with increasing temperature and stress. The presence of a pre-oxidized oxide layer can increase the creep resistance and reduce the creep rate; however, the high stress (>10 MPa) is sufficient for peeling the oxide layer and reducing the resistance of the oxide layer (Figure 6). Additionally, the creep stress strips the original oxide layer, causes the deformation of the metal lattice, accelerates the ionic mobility, improves the kinetic rate of metal oxidation, and promotes the further oxidation of the new metal region [48].

The most important factor affecting the creep of the cell during operation is stress. Therefore, ferritic stainless steel, which is relatively close to the thermal expansion coefficient of other functional layers (less thermal stress) and has good oxidation resistance, is a satisfactory candidate material for support. In addition, the above-mentioned use of coatings expands the selection of anode and cathode catalyst materials on the cell skeleton, making it possible to introduce materials that do not match the thermal expansion coefficients of other functional layers.

### 2.4. Electrolyte Defects

**Figure 7:**
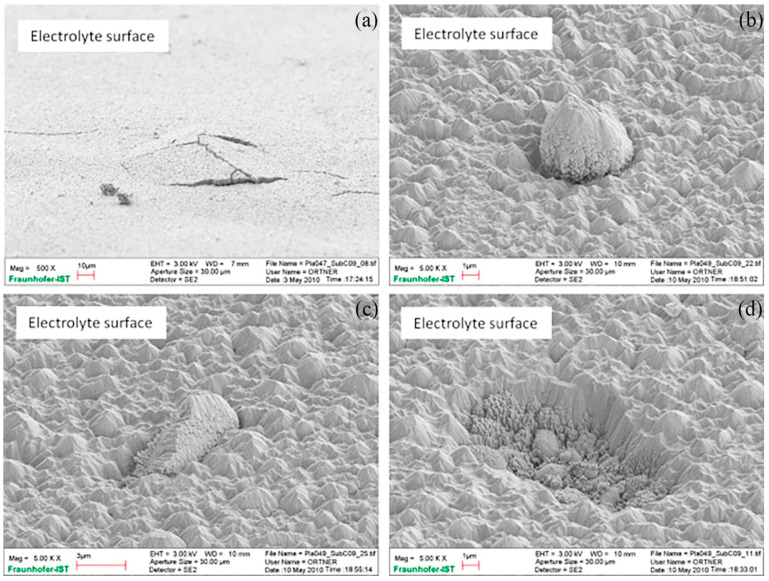
Typical electrolyte coating defects: (**a**) bulges and spallation, (**b**) nodular growth, (**c**) accretion growth, and (**d**) outburst. Note the different magnifications of (**a**–**d**) [52].

The relatively inexpensive and high-strength metal support can provide sufficient mechanical properties for the cell so that the thickness of each part of the SOFC (especially the electrolyte as the core component) is reduced to reduce the ohmic impedance, improve the performance, and reduce the cost. However, traditional ceramic preparation technology has difficulty preparing electrolytes with thicknesses of less than 10 μm, seriously limiting the improvement in performance; physical and chemical vapor deposition and other thin film preparation technologies can achieve this phenomenon. With the development of thin-film technologies, the thickness of the electrolyte can be as low as the submicron level, and the operating temperature can be as low as 450–550 °C [50,51]. In addition, these low-temperature film technologies can avoid performance degradation caused by high preparation temperature. However, the recent spraying technology is still not sufficiently advanced; even if the electrolyte thickness can be thinned, it is difficult to achieve the long-term stable operation of the MS-SOFC. Certain defects, such as the interface bonding of adjacent layers, microcracks, and nodules of the electrolyte, have seriously limited the stability of the cell (Figure 7); further technical optimization is still needed in the future.

## 3. Optimization

To solve the problems in MS-SOFCs, in addition to innovating materials and technologies, researchers have mainly adopted pre-oxidation treatment, introduced a barrier layer and coating, and performed precoarsening treatment [20,28,29,40]. Among these options, the coating prepared at low temperature can effectively avoid the coarsening of the catalyst of the traditional high-temperature cosintered sandwich structure SOFC, improve the oxidation resistance, reduce cation interdiffusion and toxicity, and make it possible to introduce materials with mismatched thermal expansion coefficient, which is a comprehensive optimization technology with significantly improved overall performance [29,39,40,41,53].

High temperature are known to accelerate the failure of MS-SOFCs. Traditional high-temperature cofiring techniques, such as casting and screen printing, accelerate oxidation, diffusion, coarsening, and other problems during cell preparation; thus, it is necessary to improve the technology to enable it to be prepared at low temperature [32,33]. To date, common low-temperature preparation technology includes various techniques, including the infiltration method, spraying method, and sintering aids method.

### 3.1. Infiltration Method

The impregnation of MS-SOFC is based on the existing cell bone structure. The required metal salt is mixed into a solution, and impregnation, standing, drying, low-temperature sintering, and other operations are performed to form the required impregnation coating layers. For the impregnated porous support in MS-SOFC, coating layers of the impregnated technology are only distributed on the support surface in most cases, greatly increasing the contact areas between the reactant molecules and maintaining the porous shapes of the support and functional layer. The continuous coating formed by calcination at low temperature can reduce the problems of oxidation, mutual diffusion, and coarsening of the catalyst at high temperature and solve the thermal mismatch between the support and adjacent functional layers. The feasibility of this impregnation technique with a high utilization rate, low consumption level, simple operation ability, and low cost is clear. Researchers have conducted much research and technical improvement on it, which will be mainly described below [54,55,56,57,58,59,60,61,62,63,64].

**Figure 8:**
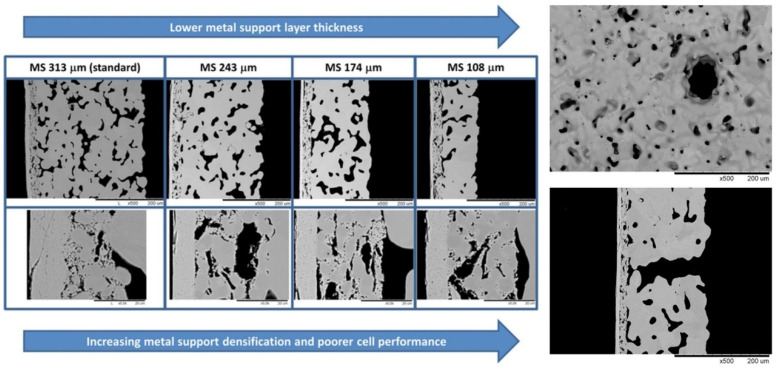
Half-cells with systematically varying metal support thickness and the introduction of the gas channel in SEM images [55].

Impregnation technology is innovated based on the pore structure, surface quality, other micromorphologies of the support and impregnation parameters. The results show that the microstructure characteristics of the metal support, such as the pore size, distribution, and surface quality, have certain influences on the deposition and distribution of the impregnated layer. After impregnation, cells with open microstructures have uniform coating distributions and low surface-specific resistance at the support and functional layer [54]. Notably, to reduce the volume and weight of the cell stacks, reductions in the thickness of the support, which occupies most of the volume in MS-SOFCs, are often adopted to make the cell suitable for use in mobile applications, but this increases the densification of the cosintered support and decreases the performance. Nielsen et al. [55] have found that the cell performance can be improved by introducing gas channels by puncturing the support and increasing the porosity of the support by reducing the dwell time (Figure 8). In addition, a narrower pore size distribution makes evaporation more uniform, reduces the promoting effect of capillary force on large deposition in small pores during heating evaporation, and obtains an evenly distributed impregnation layer [56]. For insufficient capillary wetting force, vacuum treatment and solvent-assisted vacuum-free processes can help precursors impregnate pores [57]. In addition, the effectiveness of the impregnation layer depends on the surface characteristics of the metal support, affecting the quality of the initial coating and the subsequent oxidation behavior. The electronic conductivity of the alloy support is very important for promoting rapid hydro-oxidation kinetics and obtaining low-polarization-resistance coated electrode [58]. Stefan et al. considered that the surface of the clean metal support is highly hydrophobic, while the metal surface with a trace of silicon oxide pollution is hydrophilic; an appropriate impregnation treatment can be selected according to the requirements [59].

The results show that different parameters, such as the dilution of the precursor, number of coating layers, catalyst load, and catalyst calcination temperature, affect the coating quality. To obtain excellent coating performance, it is often necessary to conduct specific analyses and tradeoffs according to the characteristics of the required materials. Because the viscosity of the high-concentration molten salt precursor is quite high, when the melting temperature of the precursor is near the impregnation temperature, it is more suitable to dilute the precursor into a solution with low viscosity and low surface tension that can be stored for several months and remain liquid in a wide range of temperature for impregnation operation; this phenomenon prevents crystallization during impregnation and helps realize the industrial production scale [60]. However, diluting the precursor results in less material being deposited in each impregnation cycle; thus, more impregnation cycles are required to achieve the desired load to prevent too little load from forming a continuous layer. Notably, the increasing number of layers does not always lead to improved results. Too many layers will make the load too high and block gas transmission. Stange et al. [61] found that the more coating layers, the higher the cracking degree of La(Mn_0.5_Co_0.5_)_0.8_ coating, which can be coated up to five times. This is because replacing Mn with Co in LaMnO_3_ will improve the TEC of the coating (increasing the difference from the support) [61]. The specific number of impregnation layers needs to be adjusted according to coating characteristics, requirements, and experiment situation [61,62]. In addition, to make the coating more continuous and to increase the specific surface area, different heat treatments can be applied to each layer [60,62]. Tucker et al. [60,62,63] found that a high primary impregnation calcination temperature can improve the sintering connection among particles and improve electronic conductivity, and a low subsequent impregnation calcination temperature can provide a high specific surface area. The impregnation of the anode and cathode at a high temperature for the first time significantly improved the performance [60]. In addition, to reduce the time of each impregnation cycle, avoid the oxidation of the support (the traditional impregnation heat treatment cycle requires 5–7 h), and increase the feasibility of industrial production, the rapid impregnation technology of directly placing the impregnation cell into the preheating furnace, short-term reaction (approximately 10 min), and removing the air cooling can be used [61,64]. This rapid conversion reaction from nitrate to oxide can remove the excess catalyst in the pore and improve mass transport [64]. In conclusion, to obtain the best cell performance, the impregnation process parameters must be balanced comprehensively.

Large quantities of impregnation materials have been studied, some of which are shown in Table 1.

Different impregnation materials can be selected for coating introduction. SFMO catalyst material avoids the problem of Ni agglomeration [41], 5 wt.% Rh-CZ improves the carbon deposition resistance of the cell [74], the La(Mn_0.5_Co_0.5_)_0.8_ protective coating significantly improves the oxidation resistance [61], and the nanostructure Ni-GDC infiltrating anode has a significantly high tolerance to sulfur poisoning [54]. To ensure the stability of the coating, it is sometimes necessary to limit the operating temperature of the MS-SOFC. When using copper-based coating, it is likely necessary to limit the operating temperature to below 650 °C; the support covered by the La_0.2_Sr_0.8_Ti_0.9_Ni_0.1_O_3−δ_ (LSTN) coating should be used below 680 °C [75,76]. However, this process is acceptable for MS-SOFCs used primarily at lower temperature.

In conclusion, to improve the coating morphology, different impregnation materials and treatments can be selected in various cycles. First, while ensuring the mechanical properties of the cell, the porosity of the cell should be improved as much as possible. For the first impregnation, an antioxidant material with good conductivity is used for vacuum impregnation, and the coating is quickly calcined at a high temperature to ensure the continuity of the coating. According to the performance requirements, specific materials are selected, and the vacuum impregnation, and low-temperature rapid calcination are carried out, the subsequent impregnation provides a high catalyst surface area. The specific impregnation parameters must be adjusted through experiments, and the operating temperature of the cell should be limited. Notably, this idea is temporary, mainly because a catalyst material with high oxidation resistance, coarsening resistance, carbon accumulation resistance, and poison resistance has not yet been developed. To date, SDCN/PrO_x_-impregnated cells with excellent performance still have high price and steep performance decline after precoarsening. In the future, we must further research and develop impregnation materials [62].

### 3.2. Thermal Spraying Method

Thermal spraying, which is a promising alternative to wet ceramic processing, is a process in which spray materials in a molten or solution state are sprayed on the surface of a sample by high-speed airflow to form a spray layer [77,78,79,80,81,82,83,84]. The low operating temperature can restrain the high-temperature oxidation, the coarsening of the catalyst, and interdiffusion during cell preparation to a great extent. In addition, spraying technology is very suitable for preparing thin functional layers, especially dense thin electrolyte layers [50,51]. The reduction in thickness can effectively reduce the ohmic impedance and improve the MS-SOFC performance. However, the spraying method and parameters for preparing each functional layer must be adjusted accordingly. To optimize the morphology of the spraying layer (especially the spraying electrolyte layer), the sprayed cell is often subjected to low-temperature heat treatment (relative to the traditional cofiring temperature) to increase stability.

To date, the technologies that have been used for MS-SOFC processing include atmospheric plasma spraying (APS), very-low-pressure plasma spraying (VLPPS), suspended plasma spraying (SPS), the aerosol deposition process (ADP), etc. APS technology is used to prepare functional and electrolyte layers on Ni-Mo, Ni-Fe, and Ni-MoFe porous supports, and MS-SOFCs without cracking and deformation are finally obtained [77,78,79]. The durability of the Ni-MoFe-supported SOFC is relatively poor (approximately 20% kh^−1^ in the durability test at 650 °C and 280 h); however, the performance of the Ni-MoFe-supported SOFC can be effectively restored by heat treatment. Due to the lack of airtightness in electrolytes prepared by the APS method, researchers have used a nitrate solution to permeate the coating and increase the preheating temperature of the substrate to improve the densities of electrolytes [80,81]. In addition, VLPPS is a promising method for preparing dense electrolyte coatings. When the ambient pressure is low, the plasma flow rate expands greatly, helping to increase the particle velocity and temperature, thus improving the internal adhesion of the ceramic coating. Although there are some tiny cracks and voids, the interface bonding characteristics between the adjacent layers are improved, and the conductivity of the electrolyte layer is increased [82]. Li et al. used VLPPS technology to prepare a uniform and dense electrolyte layer, and the maximum power density reaches 1164 mW cm^−2^ at 750 °C [83]. Moreover, SPS technology can realize the mixing of immiscible phases, and the prepared LSCF–GDC composite cathode does not require any postdeposition sintering; this phenomenon can avoid the excessive oxidation of the metal support during air sintering and the degradation of perovskite materials during sintering at low oxygen partial pressures [84]. The area-specific resistance (ASR ≈ 1.50 Ω cm^2^) of the LSM/8YSZ composite cathode prepared by the ADP technique at 800 °C is significantly lower than that of the samples usually reported to be prepared in situ [85].

MS-SOFCs with thin-layer electrolytes prepared by the spraying method are a promising development direction. However, the thin electrolyte thickness has high requirements for preparation. This thin layer magnifies various errors and defects in preparation, which can easily make the cell fail. The defects and particle impurities on the anode surface or the anode itself seriously affect the airtightness of the electrolyte layer [52]. The finer anode structure facilitates the preparation of the electrolyte layer. In addition, the deposition of an electrolyte structure with alternating reactive oxidic and metallic layers can break the columnar grain structure of the monolayer electrolyte and significantly improve the airtightness of the electrolyte and the performance of the cell. The current density of the cell with multiple electrolytes exceeds 2 A cm^−2^ at 850 °C and 0.7 V [52]. Most existing thin-layer electrolyte cells have poor long-term performance and must be further developed.

In recent years, the power density and long-term stability of sprayed MS-SOFCs have been continuously enhanced; however, their durability still must be further improved, especially for thin-layer electrolyte MS-SOFCs [52]. Additionally, the production costs must be further reduced to realize industrial production.

### 3.3. Sintering Aids Method

The densification temperature of SOFC electrolyte prepared by the traditional cosintering method is very high (1350–1600 °C), and there are high requirements for the equipment [6,7,86,87,88,89]. In addition, when matching with other functional layers, due to the mismatch of the thermal expansion coefficient and sintering rate, electrolyte density deficiency and cell breakage often occur [90]. Moreover, it is sometimes difficult for the metal support in MS-SOFCs to withstand high sintering temperature [88,89]. These problems bring great trouble to the fabrication of cells. Adding a small amount of sintering aids to the electrolyte material can adjust the coefficient of thermal expansion, improve the densification degree, effectively reduce the densification temperature of the cells, and reduce the adverse influence of high-temperature cosintering during fabrication [91,92,93,94,95,96,97,98].

Sintering aids are oxides or nonoxides that promote the densification of ceramics. To date, common SOFC electrolyte sintering aids include Fe_2_O_3_, Co_3_O_4_, CuO, Al_2_O_3_, Li_2_O, etc. [91,92,93,94,95]. Ishihara et al. [88] conducted research on LSGM electrolytes first. Chang et al. [95] used Li_2_O sintering aids in LSGM electrolytes of Ni-Mo-supported cells. On the one hand, the addition of Li_2_O can improve the thermal expansion coefficient of LSGM (12.4 × 10^−6^ °C^−1^), making it better matched with the Ni-Mo support (14.5 × 10^−6^ °C^−1^). On the other hand, an intergranular liquid phase can be formed on the surface of LSGM grain boundaries to reduce the friction between particles, accelerate the movement of grain boundaries, promote grain growth, and increase electrolyte density [95]. In addition, 1 wt.% Al_2_O_3_ can reduce the sintering temperature of the YSZ electrolyte to 1250 °C, and 1 mol% Co_3_O_4_ can reduce the sintering densification temperature of GDC to 900 °C [92,94]. The Co_3_O_4_-doped GDC electrolyte layer is fabricated by Ceres Power in the UK by screen printing on a laser drilling stainless steel sheet; this material can be sintered in air at approximately 1000 °C and has an OCV of 1.10 V at 570 °C [96].

Although studies have shown that the introduction of sintering aids can reduce the ionic conductivity of the electrolyte, its performance is expected to be improved due to the densification of the electrolyte [91]. Gao et al. found that the SOFC performance of all iron-doped YSZ electrolytes are better than those of noniron-containing electrolytes [97]. In addition to density and conductivity factors, this phenomenon may be related to the improvement in the electrolyte–electrode interface by doping iron, and the specific mechanism still needs further study [97]. Notably, the sintering aids can be added to other functional layers to promote sintering. Adding 5 wt.% CuO can reduce the sintering temperature of the Er_0.4_Bi_1.6_O_3−δ_—La_0.8_Sr_0.2_MnO_3−δ_ (ESB–LSM) cathode to 650 °C to avoid oxidation of the support during sintering [98]. To date, there are few studies on sintering aids in the field of MS-SOFCs, and further research is needed.

In summary, three low-temperature technologies can be integrated, including a thin-layer deposition of a sintered aid-doped electrolyte to reduce electrolyte defects and improve cell durability. When impregnating the electrode skeleton, sintering adds can be added in the first impregnation cycle to improve the coating continuity and reduce the preparation temperature. The low operating temperature of the thin-layer electrolyte cell contributes to the long-term stable operation of the impregnated cell.

## 4. Conclusions and Outlook

Metal-supported solid oxide fuel cells (MS-SOFCs) have good commercial prospects. However, MS-SOFC development still faces many challenges, including high-temperature oxidation, cation interdiffusion, heat matching, and electrolyte defects. These challenges limit the long-term stabilities of MS-SOFCs and seriously hinder their commercialization process. Optimization strategies that have been used to address the problems in MS-SOFCs include pre-oxidation treatment, barrier layer introduction, coating introduction, and precoarsening treatment. Among these strategies, the introduction of the coating is a comprehensive optimization strategy that can significantly improve the overall performance.

High temperature is the main cause of the failure of MS-SOFCs. To slow the failure of cells, it is necessary to further develop low-temperature preparation technologies, such as the impregnation method, spraying method, and sintering aids method. When using the impregnation method, in order to improve the stability and compatibility of cells, different impregnation treatments should be selected in different impregnation cycles according to the requirements. To ensure the stability of the coating, the operating temperature of the cell must be limited. To date, various spraying technologies have been successfully applied to MS-SOFCs, and the compatibility of cells has been improved through technological innovation, so as to achieve good performance, but the stability of the functional layer still needs to be improved, and the production cost still needs to be reduced. The addition of sintering aids can reduce the preparation temperature of each functional layer, improve the matching of each layer, and even break the requirement of traditional stainless steel supported cell sintering in a reducing atmosphere, improving the safety of the experiment.

Although some achievements have been made to prepare MS-SOFCs with low-temperature technology, there is still a large space for development. To date, it is still difficult to produce MS-SOFCs with high output power, good stability, low price, and simple and fast processing. On the one hand, there are still great limitations of existing materials, and electrode and electrolyte materials with excellent output performance are expensive and lack stability; thus, new materials still need to be researched and developed. However, due to the difficulty in the research and development of new materials, the structural optimization of existing materials can be started. Structural optimization, such as core–shell structure and heterogeneous structure, can improve the electrical efficiency and stability of cells. On the other hand, the recent production technology is not sufficiently mature, and there are many problems, such as complicated processes, high costs, and many defects in preparation, which are not suitable for industrial large-scale production and need further technological innovation. By adjusting the cell structure design and preparation process parameters, the morphology of the functional layer can be carefully controlled to reduce the preparation defects. Integrating various existing preparation processes can reduce the inherent problems of a single process. In summary, to solve the failure problem of MS-SOFCs and achieve commercialization, it is necessary to further design cell materials and structures, and continue to develop the low-temperature preparation of MS-SOFCs.

## Figures and Tables

**Table 1:** Summarized MS-SOFC parameters with various catalyst compositions.

Cell Substrate	Impregnation Material	Fuel Gas Composition	Operating Temperature (°C)	Open-Circuit Voltage (OCV) (V)	Peak Power (W cm^−2^)	Durability	Year	Reference
Fe-22Cr/Fe-22Cr + YSZ/ScYSZ/LSCF-GDC/LSC	Ni-GDC/-	H_2_-4%H_2_O	650	~1.11	~0.43	<~5% kh^−1^	2011	[65]
430 L/YSZ/YSZ	Ni/SSC	H_2_-3%H_2_O	750	1.0	0.38	Dropped by 64% (12 h)	2013	[66]
	SFMO/SFMO		800	~1.07	0.81	-	2014	[58]
	Ni-SDC/LSFSc		600	1.12	0.40	No obvious degradation (190 h)	2014	[67]
430 L/430 L-YSZ/SSZ/SSZ	Ni-SDC/SBSCO	H_2_-3%H_2_O	700	~1.10	1.25	-	2014	[68]
430 L/SSZ/SSZ	Ni-SDC/LSFSc	H_2_-3%H_2_O	650	~1.10	0.53	1.3% kh^−1^	2015	[69]
430 L/SSZ/ESB-Ag-LBSM	Ni-SDC/-	H_2_-3%H_2_O	600	~1.10	0.46	No obvious degradation (90 h)	2015	[70]
FeCr/LSFNT-FeCr-ScYSZ/ScYSZ/GDC/LSC	Ni-GDC/-	H_2_-20%H_2_O	750	~1.03	1.07	-	2017	[56]
P434L/YSZ/YSZ/YSZ/P434L	SDCN/LSM	H_2_	700	~1.10	~1.20	60% kh^−1^	2017	[71]
	SDCN/SDCN			~1.12	~0.60	54% kh^−1^		
P434L/SCSZ/SCSZ/SCSZ/P434L	SDCN/LSM	H_2_-3%H_2_O	700	1.12	0.9	-	2019	[72]
	SDCN/LSF			1.09	0.7			
	SDCN/LSC			1.09	1.0			
	SDCN/LSCF			1.10	0.8			
	SDCN/SSC			1.10	1.0			
	SDCN/PrO_x_			1.12	1.30			
	2xNi-SDCN _40_/PrO_x_	ethanol	700	~1.03	1.40	-	2020	[73]
		ethanol-water		1.02	1.32	No carbon deposition		
430 L/ScYSZ/ScYSZ/LSC	5 wt.% Rh-CZ/-	ethanol	600	1.0	0.15	1.5 mV h^−1^ (130 h)	2022	[74]

Abbreviations: 22% Cr-based stainless steel alloy (Fe-22Cr); Yttria-stabilized zirconia (YSZ); ZrO_2_ co-doped with Sc_2_O_3_ and Y_2_O_3_ (ScYSZ); lanthanum strontium cobaltite (LSC); Sm_0.5_Sr_0.5_CoO_3_(SSC); SrFe_0.75_Mo_0.25_O_3−δ_ (SFMO); La_0.6_Sr_0.4_Fe_0.9_Sc_0.1_O_3−δ_ (LSFSc); scandia-stabilized zirconia (SSZ); SmBa_0.5_Sr_0.5_Co_2_O_5+δ_ (SBSCO); (Bi_2_O_3_)_(0.7)_(Er_2_O_3_)_(0.3)_-Ag (ESB-Ag); La_0.74_Bi_0.10_Sr_0.16_MnO_3−δ_ (LBSM); La_0.4_Sr_0.4_Fe_0.03_Ni_0.03_Ti_0.94_O_3_ (LSFNT); lanthanum strontium ferrite (LSF); rhodium–CeO_2_–ZrO_2_ (Rh-CZ).

## Data Availability

Data sharing is not applicable to this article.
